# Zinc Oxide Nanowires Exposure Induces a Distinct Inflammatory Response via CCL11-Mediated Eosinophil Recruitment

**DOI:** 10.3389/fimmu.2019.02604

**Published:** 2019-11-08

**Authors:** Ruqaih S. Alghsham, Shuchismita R. Satpathy, Sobha R. Bodduluri, Bindu Hegde, Venkatakrishna R. Jala, Waleed Twal, Joseph A. Burlison, Mahendra Sunkara, Bodduluri Haribabu

**Affiliations:** ^1^Department of Microbiology and Immunology, University of Louisville, Louisville, KY, United States; ^2^James Graham Brown Cancer Center, University of Louisville, Louisville, KY, United States; ^3^Regenerative Medicine and Cell Biology, Medical University of South Carolina, Charleston, SC, United States; ^4^Department of Chemical Engineering, Conn Center for Renewable Energy, University of Louisville, Louisville, KY, United States

**Keywords:** engineered nanomaterials, zinc oxide nanoparticles, zinc oxide nanowires, murine model, toxicity, lung inflammation

## Abstract

High aspect ratio zinc oxide nanowires (ZnONWs) have become one of the most important products in nanotechnology. The wide range applications of ZnONWs have heightened the need for evaluating the risks and biological consequences to these particles. In this study, we investigated inflammatory pathways activated by ZnONWs in cultured cells as well as the consequences of systemic exposure in mouse models. Confocal microscopy showed rapid phagocytic uptake of FITC-ZnONWs by macrophages. Exposure of macrophages or lung epithelial cells to ZnONWs induced the production of CCL2 and CCL11. Moreover, ZnONWs exposure induced both IL-6 and TNF-α production only in macrophages but not in LKR13 cells. Intratracheal instillation of ZnONWs in C57BL/6 mice induced a significant increase in the total numbers of immune cells in the broncho alveolar lavage fluid (BALFs) 2 days after instillation. Macrophages and eosinophils were the predominant cellular infiltrates of ZnONWs exposed mouse lungs. Similar cellular infiltrates were also observed in a mouse air-pouch model. Pro-inflammatory cytokines IL-6 and TNF-α as well as chemokines CCL11, and CCL2 were increased both in BALFs and air-pouch lavage fluids. These results suggest that exposure to ZnONWs may induce distinct inflammatory responses through phagocytic uptake and formation of soluble Zn^2+^ ions.

## Introduction

In the past decade, use of the nanotechnology has become widespread. The rapid development of engineered nanomaterials have created a whole range of new forms of the nanoparticles with different sizes and shapes (1). Engineered zinc oxide (ZnO) nanoparticles (NPs) are widely used in different applications and consumer products such as anti-dandruff products, baby powders, sunscreens, and cosmetics (2, 3). A new class of high aspect one-dimensional (1D) ZnO nanowires (ZnONWs) have recently been developed (4). Due to their unique optoelectronic properties, these ZnONWs have important applications in the development of nanosensors, photocatalysts, nano-transducers (4), and heterogeneous catalysis (5). Recent results indicate that vapor phase production methods could potentially be utilized for commercial scale production of ZnONWs (5). In biological systems, Zinc plays an important role in the physiological processes as it supports the function of more than 300 enzymes in the body (6). Therefore, Zinc deficiency can lead to growth impairments in children and increases the risk of infection (7). On the other hand, excessive zinc consumption can have a variety of adverse effects such as gastric pain, neurodegenerative conditions like Alzheimer's and brain damage (8, 9). Additionally, inhalation of zinc oxide fumes causes metal fume fever syndrome among foundry workers (10). Thus, the increase in production and use of ZnONWs may pose a significant risk of exposure to public and workers.

Based on the potential toxicity of many engineered nanomaterials (ENMs) the National Organic Standards Board issued a recommendation for banning ENMs in food products (11). Previous studies have assessed the cytotoxicity of ZnONPs *in vitro* in a variety of mammalian cell lines such as macrophage, liver, and lung epithelial cells (12–14). The toxicity is mainly due to the soluble Zn ions generated in the acidic environment of the phagolysosomal compartment of the cell, leading to increased concentration of free Zn ions inside the cell. Autophagy was suggested as a possible mechanism in which ZnONPs induce toxicity and cell death as a result of reactive oxygen species (ROS) production (15). Furthermore, *in vivo* studies showed that intra-tracheal instillation of ZnONPs causes lung inflammation in mice (16, 17). In addition, ZnONPs were shown to induce eosinophilia in a murine asthma model (18). ZnONWs share the same chemical composition as the ZnONPs. However, very limited information is available on the toxicity and the underlying mechanisms of the ZnONWs induced inflammation. Since the shape is a factor that contributes to the toxicity of the ENMs, ZnONWs may elicit a distinct inflammatory response from ZnONPs.

In this study, our objective is to establish the toxicity and inflammatory potential of the ZnONWs. Confocal microscopy showed that ZnONWs remained as particles only in cells exposed to bafilomycin-A1 (Baf-A1), an inhibitor of vacuole acidification (19). Exposure of cultured bone marrow derived macrophages (BMDM) to ZnONWs resulted in upregulation of inflammatory cytokines IL-6 and TNFα. We show that exposure to ZnONWs induced the recruitment of macrophages and eosinophils in lung and air-pouch models in C57BL/6 mice. Consistent with the macrophage and eosinophil recruitment, CCL2, and CCL11 are the predominant chemokines in bronchoalveolar lavage fluids (BALFs) from ZnONWs treated mice. Both IL-6 and TNF-α were also upregulated in BALFs from the ZnONWs treated mice. Similar cytokine and chemokine profile was also observed in air pouch lavage fluids. These studies contribute to the understanding of the potential mechanisms involved in ZnONWs induced inflammation.

## Materials and Methods

### Mice

C57BL/6 mice were purchased from Jackson Laboratories. All mice were sex and age matched 6–8 weeks old. Mice were maintained in a specific pathogen free (SPF) facility and all the procedures were approved by University of Louisville Institutional Animal Care and Use Committee (IACUC).

### Nanoparticles and Reagents

Zinc Oxide nanoparticles ZnONPs (10–30 nm) and ZnONWs (100 nm) were provided by Advanced Energy Materials, LLC, a nanowire powder manufacturing company in Louisville, KY. Silicon dioxide nanoparticles (SiO2NPs 7 & 200 nm) were obtained from sigma Aldrich. All particles were made endotoxin-free by baking at 200°C overnight. The nanowires were heated up to 200°C slowly with gradual increase of 5°C/min to prevent structural alterations. The particles were characterized for size and morphology by scanning electron microscopy (SEM) and for chemical composition by energy dispersive X-ray Spectroscopy (EDX). The data (Supplementary Figure 1) shows the size, morphology and chemical composition did not change significantly after heat inactivation. The particles were resuspended in 1xPBS fresh for each experiment, vortexed, and added to cells immediately to minimize aggregation. All experiments in this study used only baked material and the ZnONWs as prepared were never used in biological experiments. Endotoxin levels were measured in original and the baked samples using Limulus amebocyte lysate assay (Chromogenic LAL) (20). The level of endotoxin contamination of ZnONWs was 0.863(EU)/ml before baking, whereas the levels of endotoxin after baking was a significantly lower at 0.18 (EU)/ml. The following pharmacological inhibitors were used in the study: Cytochalasin D (from Sigma-Aldrich) Bafilomycin-A1 (Santa Cruz). LPS (LPS-EK; InvivoGen).

### FITC-Labeling of ZnONWłs

Four mg of ZnONWs were dispersed in 3 ml anhydrous dimethylformamide (DMF). Then a diluted solution of 0.5 μl of amino-propyl-triethoxy-silane (APTS) in 25 μl DMF was added to the ZnONWs suspensions, which was followed by sonication, and stirring under nitrogen at room temperature for 20 h. ZnONWs were collected by centrifugation and removing the supernatant. After washing, ZnONWs were re-suspended in 0.5 ml DMF and mixed with a solution of 1 mg FITC and 0.5 ml DMF. The suspension was stirred for 4 h, and the FITC-labeled ZnONWs were collected by centrifugation. FITC-labeled ZnONWs were thoroughly washed with DMF, dried under vacuum and stored as dry powders (21).

### Cell Culture

RAW 264.7, mouse macrophage cell line (American Type Culture Collection (ATCC), Manassas, VA, USA) and LKR13 cells, murine K-ras mutant lung adenocarcinoma cell line were maintained in Dulbecco's Modified Eagle's Medium (DMEM; Hyclone Laboratories, Inc., South Logan, UT, USA). DMEM was supplemented with 10% heat-inactivated fetal bovine serum (FBS, Hyclone Laboratories Inc.) and 100 units/ml Penicillin, 100 μg/ml Streptomycin, 2 mM L-Glutamine, and 50 μM β-mercaptoethanol (Gibco®, Invitrogen Corporation, Carlsbad, CA, USA) at 37°C in a 5% CO_2_ incubator. Cells were seeded at 0.5 × 10^6^ cells/well density into six well plates.

### Bone Marrow Derived Macrophages

Six to eight-week-old C57BL/6 mice were euthanized using CO_2_. The hind legs were dissected and the bone marrow cells were flushed out. The bone marrow cells were cultured in Dulbecco's Modified Eagle Medium (DMEM) containing 10% FBS,100 units/ml penicillin, 100 mg/ml streptomycin, 2 mM L-glutamine, and 50 mM β -mercaptoethanol supplemented with 25 ng/ml recombinant mouse macrophage colony stimulating factor (BioLegend; San Diego, CA). The cells were plated at a density of 1 million cells per 100-mm tissue culture dishes containing 10 ml of medium. On day 3, 10 ml of fresh growth medium was added to replace the medium. The cells were maintained for another 3 days before the experiments.

### *In vitro* Stimulation With ZnONWs

BMDMs, RAW264.7 cells or LKR13 cells were plated at a density of 0.5 × 10^6^ cells per well in six well tissue culture plates (VWR) in 2 ml of Hyclone DMEM containing 10% FBS and allowed to attach overnight. Cells were primed with and without 10 ng/ml of LPS (LPS-EK; InvivoGen, San Diego, CA) for 3 h. After washing with 1X PBS three times the cells were changed to 1 ml 1% FBS media for stimulation. In some experiments the cells were then pre-treated with or without the pharmacological inhibitors Baf-A1 or CytD at the indicated concentrations for 1 h prior to stimulation with 10 or 20 μg/ml of particles for 3–6 h as indicated. At the indicated times the cell culture supernatants were collected centrifuged and stored at −80°C for subsequent ELISA analysis.

### Intratracheal Instillation of Particles

Mice were treated with antibiotics for 1-week prior to surgery. Mice were surgically instilled (intratracheal) with endotoxin-free PBS (negative control) or 0.67 mg of endotoxin-free SiONPs (positive control) or 0.67 mg of ZnONWs suspended in endotoxin-free PBS. Previous studies have reported the use of 0.2 μg/mouse (22) and 0.168 μg/mouse of ZnONPs (23). Particles suspension was vortexed before instillation to avoid settling of the particles. Mice were continuously maintained on antibiotics until euthanasia 2 days after instillation. Lungs were lavaged with 2 ml of cold PBS and BALF were obtained.

### Air Pouch Model

Five ml of sterile air was injected into the back of mice subcutaneously to generate an air pouch. Three days later another 3 ml of sterile air was injected into the pouch to maintain the air-pouch. After 3 days, 2 mg of particles in 500 μl of endotoxin-free PBS was injected into the air pouch. Control animals received 500 μl of endotoxin-free PBS. After 6 h of injection, mice were euthanized and the air pouch was lavaged with 3 ml of cold PBS. Using Shandon Cytospin centrifuge (Shandon Lipshaw) the air pouch lavage fluid cells and BALFs cells were spun down. Slides were stained with Hema-3 reagents (ThermoFisher Scientific) according to the manufacturer's recommendations.

### Identification of Immune Cells by Flow Cytometry

Cells from air-pouch and BALFs were stained with various cell surface marker antibodies from BD Biosciences (San Diego, CA) or Biolegend (San Diego, CA). Data were acquired on FACS Calibur or FACS Canto (BD Biosciences) and analyzed using Flowjo software (Tree Star). In lungs leukocytes were identified as CD45^+^ cells, macrophages as CD45^+^ CD11c^hi^ F4/80^+^ cells, eosinophils as CD45^+^ CD11c^lo^ Siglec-F^+^ cells, neutrophils as CD45^+^ CD11c^lo^ Siglec-F^−^ Ly6G^hi^ cells. In scatter-gated cells of air-pouch lavage fluids, leukocytes were identified as CD45^+^ cells, neutrophils as Ly6G^hi^ cells, eosinophils as Ly6G^lo^ Siglec-F^+^ cells, macrophages as Ly6G^lo^ Siglec-F^−^ F4/80^+^ cells. Percentage of various cell types thus obtained were multiplied to the total cell counts for actual cell numbers of neutrophils, eosinophils, and macrophages.

### Confocal Microscopy

BMDMs were treated with Baf-A1 for 1 h, then FITC-ZnONWs were added to the cells for 2 h. Cells were fixed with 10% formalin for 15 min. Cells were washed three times with 1X PBS, followed with 594-Cholera Toxin Subunit B (ThermoFisher Scientific) and DAPI staining, and washed again before analyzing with confocal microscopy. Nikon A1 confocal microscope at 60x magnification was used to obtain images. At least five to six fields were captured.

### Enzyme-Linked Immunosorbent Assay (ELISA)

LTB_4_, IL-1β, IL-6, and TNF-α in the cell culture supernatants were measured using LTB_4_ EIA Kit (Cayman Chemicals) and Mouse IL-1β, IL-6, and TNF-α ELISA MAX Standard Kit (Biolegend), respectively, using manufacturer's instructions. Absorbance was measured using a BioTek microplate reader.

### Quantitative RT-PCR Assay

BMDMS, RAW 264.7 and LKR13 cells were lysed using TRIzol reagent and total RNA was isolated using an RNeasy Mini Kit (Qiagen) using manufacturer's protocol. RNA samples were treated with DNase (Qiagen) to remove any trace of DNA from the samples before reverse transcription with TaqMan reverse transcription reagents (Applied Biosystems) using random hexamer primers. Quantitative RT-PCR was performed using “power SYBR-green master mix” (Applied Biosystems). Expression of the target genes was normalized to GAPDH and the relative fold changes relative to the PBS were calculated using the delta CT method. Data were representative of triplicate cell cultures. GAPDH, CXCL1, CXCL2, CCL2, CCL3, CCL4, CCL5, CCL11, TNFα, and IL-6 primers obtained from Realtimeprimers.com.

### Multiplex Analysis

Lung lavage fluids and Air-pouch lavage from PBS or particles treated mice were measured for various levels of inflammatory proteins including TNF-α, CXCL1, CXCL2, CCL2, CCL4 and CCL5, CCL11, IL-1β & IL-1α. The analysis was accomplished following the standard protocols at the Proteomics core facility of Medical University of South Carolina.

### MTT Test

BMDMs, LKR13, and RAW264.7 cells were treated with ZnONWs for 2, 4, 6, or 18 h. Then 3-[4, 5-dimethyltiazol-2-yl] 2, 5-diphenyl-tetrazolium bromide (MTT) was added to the cells at the end of each time point. After 2 h incubation with MTT, DMSO was added for 10 min. and absorbance was measured at 562 nm using a BioTek microplate reader.

### Statistical Analyses

Graph Pad Prism4 Software San Diego, CA was used to analyze the Data; Data were expressed as the means ± S.E from at least three independent samples. Statistical difference among groups was analyzed using the Mann–Whitney *U*-test (*in vivo*) or Unpaired Student's *t*-test (*in vitro*/*ex-vivo*, RNA analysis). Two-tailed *P*-values of <0.05 were considered as significant.

## Results

### Preparation, Characterization and Cellular Uptake of ZnONWs

**Figure 1:**
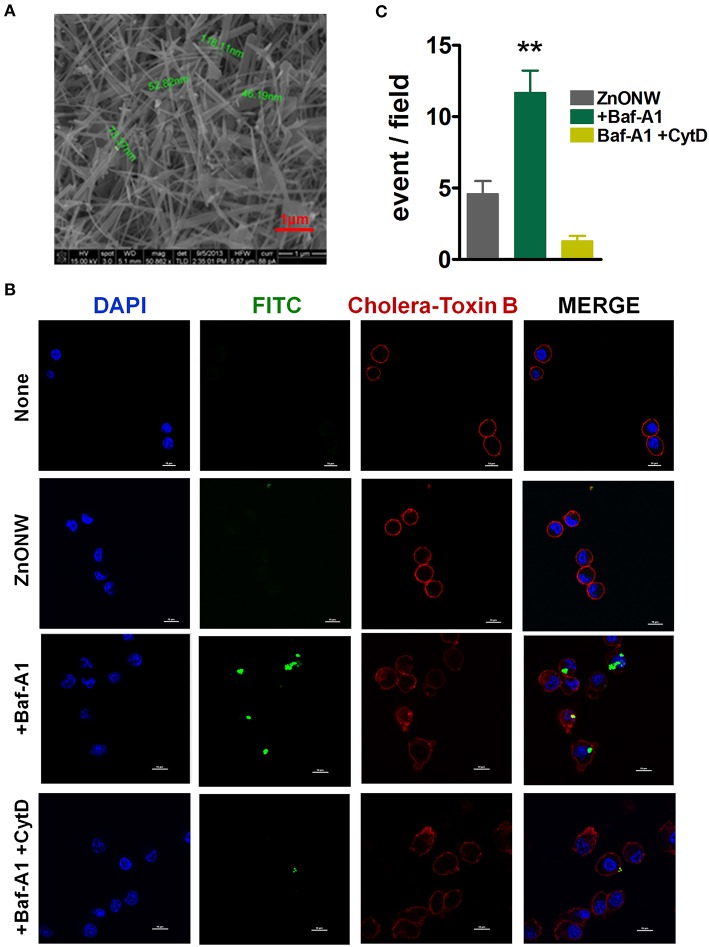
Characterization and cellular uptake of ZnONWs. Scanning Electron Microscopy (SEM) image of ZnONWs indicating size of individual nanowires **(A)**. LPS-primed BMDMs were stimulated with 25 μg/ml FITC-ZnONWs for 3 h in the presence or absence of Baf-A1 (1 μg/ml) and CytD (10 μg/ml). Cells were stained with cholera-toxin B (red) and DAPI (blue) for 20 min after fixation and visualized by confocal microscopy **(B)**. The density of intracellular ZnONWs particles was calculated as number of particle events/ high power field **(C)**. **P ≤ 0.01.

ZnONWs were synthesized by downstream microwave plasma reactor methods using rapid oxidation of zinc powders in gas phase (24). The dimensions of ZnONWs as determined by Scanning Electron Microscopy (SEM) were around 20–120 nm in diameter with 5,000 nm in length (Figure 1A). To investigate the cellular uptake of ZnONWs in BMDMs cells, FITC labeled ZnONWs were generated as previously described (21). Cells were primed with LPS for 3 h followed by 1-h pre-treatment with Baf-A1. Then cells were exposed to 25 μg/ml FITC labeled ZnONWs for 3 h, washed and examined by confocal microscopy. As shown in Figure 1B, intracellular ZnONWs were detected only in cells pre-treated with Baf-A1, whereas cells that were not treated with Baf-A1 did not show intracellular accumulation of ZnONWs particles. Furthermore, to assess the cellular uptake mechanisms of ZnONWs, cells were pre-treated with cytochalasin D (CytD) (phagocytosis inhibitor) and Baf-A1 (19, 25). CytD treated cells did not show the intracellular FITC-ZnONWs suggesting that phagocytosis is required for their uptake (Figure 1B). The extent of ZnONWs particles uptake was determined as number of particle events/high power field (Figure 1C). These results suggest that uptake of ZNONWs involves phagocytosis and that inhibition of phagolysosome formation prevents dissolution of intracellular ZnONWs in the cells.

### Cytotoxicity of ZnONWs to BMDM, LKR13 and RAW 264.7 Cells

**Figure 2:**
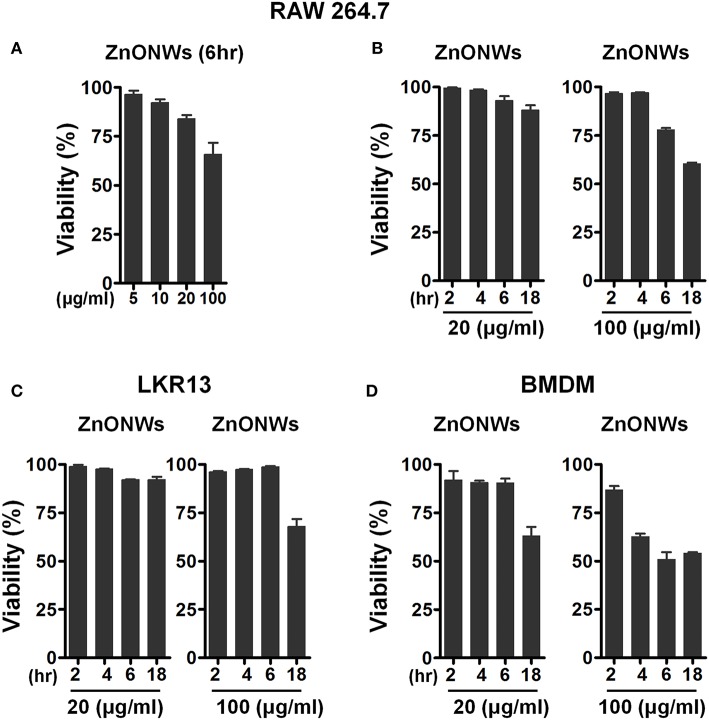
Cytotoxicity assessment of ZnONWs. BMDMs from WT mice, LKR13 or RAW-264.7 cells (0.5 × 10^6^ cells /ml) were stimulated with different concentrations for different times with ZnONWs as indicated or with PBS as negative control. Cell viability was assessed using MTT test. RAW-264.7 cells were stimulated with ZnONWs 5, 10, 20, and 100 μg/ml for 6 h **(A)**. Time course study for RAW-264.7 cells, cells were stimulated with ZnONWs 20 and 100 μg/ml concentrations for 2, 4, 6, and 18 h **(B)**. LKR13 cells were stimulated with ZnONWs 20 and 100 μg/ml concentrations for 2, 4, 6, and 18 h **(C)**. BMDMs were stimulated with ZnONWs 20 and 100 μg/ml concentrations for 2, 4, 6, and 18 h **(D)**. Data are representative of three independent experiments in triplicate cultures and values are expressed as mean ± SEM.

Previous studies showed that the ZnONPs promote toxicity in a variety of mammalian cells *in vitro*. (13, 26–28). To investigate whether the ZnONWs exhibited distinct cytotoxicity profiles, dose response and time course studies were performed. First, RAW-264.7 cells when stimulated with different doses of ZnONWs (5, 10, 20, and 100 μg/ml) for 6 h showed that at 5, 10, and 20 μg/ml doses 95% of the cells are viable whereas 100 μg/ml reduced cells viability by ~20% (Figure 2A). Thus, time course studies were performed at 20 and 100 μg/ml doses. As shown (Figure 2B), nearly 100% of the cells are viable after 18 h of treatment with 20 μg/ml dose of ZnONWs, whereas <50% of cell death was observed at 100 μg/ml. The cytotoxicity of ZnONWs was also determined in other cell types such as BMDM and LKR13 cells. In LKR13, similar results to RAW-264.7 cells were observed as shown in Figure 2C. In BMDM, we found that 20 μg/ml concentration of ZnONWs induced significant toxicity after 18 h of exposure by reduction in the cell viability up to 30%, whereas 100 μg/ml induced toxicity after only 4 h of ZnONWs stimulation (Figure 2D). Thus, all further studies with different cell types were performed at 20 μg/ml or less dose of ZnONWs for 6 h treatment.

### ZnONWs Induce Release of Pro-inflammatory Mediators by Macrophages *in vitro*

**Figure 3:**
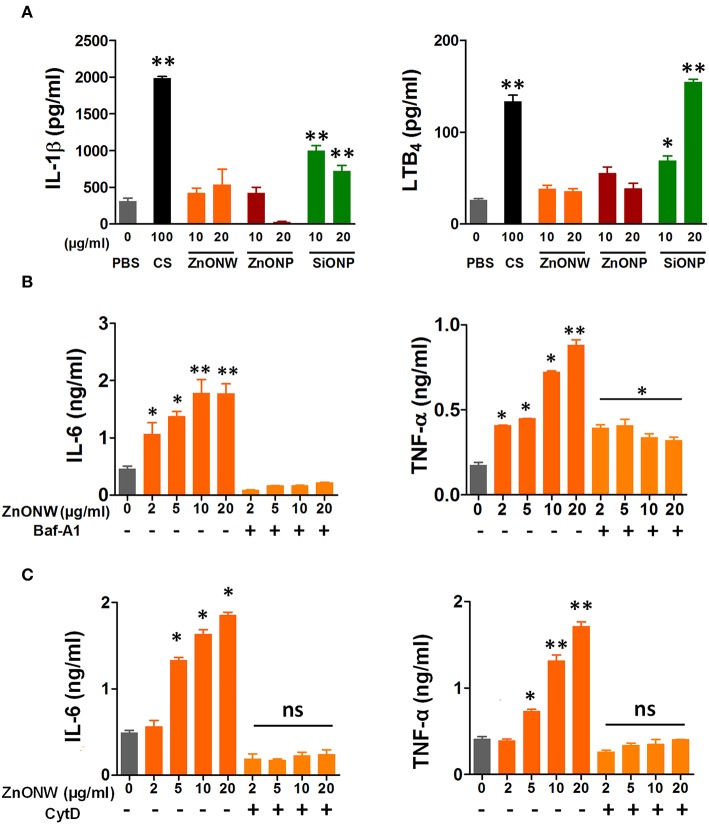
ZnONWs exposure induced release of pro-inflammatory mediators by cultured macrophages. Pro-inflammatory cytokine levels in culture supernatants from cells exposed to PBS or particles (ZnONPs, ZnONWs, and SiONPs) were analyzed using ELISA. IL-1β and LTB_4_ levels were measured in BMDMs exposed for 6 h. Cells were primed with LPS (10 ng/ml) for 3 h and washed and then treated with ZnONWs, ZnONPs, SiONPs, or PBS **(A)**. BMDMs were treated with LPS (10 ng/ml) or PBS for 3 h and washed then treated with or without Baf-A1 for 1 h. Then cells were treated with ZnONWs or PBS for 6 h, IL-6 (LPS-primed), and TNF-α (without LPS priming) levels were measured as indicated **(B)**. RAW 264.7 cells were stimulated with LPS (10 ng/ml) or PBS for 3 h and washed then treated with or without CytD (10 μM) for 1 h. Then cells were treated with ZnONWs, ZnONPs, SiONPs, or PBS for 6 h, IL-6 (LPS-primed) and TNF-α (without LPS priming) levels were measured as indicated in **(C)**. Data are representative of three independent experiments in triplicate cultures and values are expressed in ± SEM. *p ≤ 0.05, **p ≤ 0.01 non-parametric t test.

To examine the inflammatory potential of ZnONWs, BMDMs were treated with ZnONWs and the levels of pro-inflammatory cytokines such as IL-1β, IL-6, and TNF-α as well as the lipid chemokine LTB_4_ were measured in culture supernatants. Nano-silica (SiONPs) particles and ZnONPs were used as positive controls for the production of these cytokines and LTB_4_ (19, 29). Treatment of BMDMs with ZnONWs or ZnONPs did not result in any significant accumulation of IL-1β or LTB_4_ (Figure 3A). As expected, both CS and SiONPs treatments induced the production of IL-1β and LTB_4_ in BMDMs (Figure 3A). ZnONWs induced a dose dependent increase in the production of the cytokines IL-6 and TNF-α (Figure 3B). Furthermore, treatment with Baf-A1 or CytD significantly reduced the ZnONWs induced production of these cytokines (Figures 3B,C). A similar dose dependent cytokine induction profile was observed in RAW 264.7 cells treated with ZnONWs (Supplementary Figure 2). However, exposure of lung epithelial cell line LKR13 to ZnONWs did not result in either IL-6 or TNF-α production (data not shown). These results suggest that ZnONWs could induce the synthesis of pro-inflammatory cytokines IL-6 and TNF-α but not IL-1β or LTB_4_.

### Acute Skin Inflammation Induced by ZnONWs in an Air-Pouch Model

**Figure 4:**
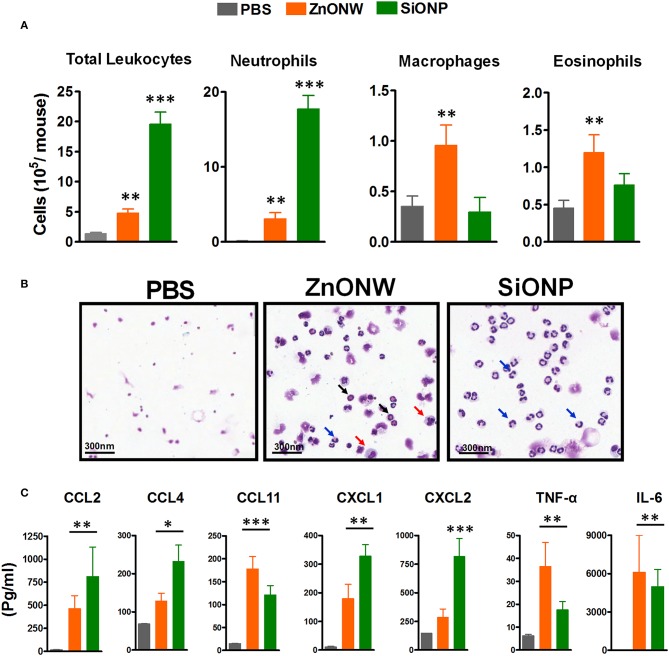
Local inflammation induced by ZnONWs in the air pouch. Sterile air was injected s.c. on the back of the wild-type (WT) mice to form an air pouch and exposed to nanoparticles (ZnONWs or SiONP) or PBS as described in methods. Six hours post particle exposure the air pouch was lavaged with 3 ml of buffer to assess infiltrating immune cells. The total number of leukocytes and differential count for cell types recruited in the air-pouch are indicated. Data are representative of at least five mice per group **(A)**. Cytospin slides showing macrophage (red arrow), neutrophils (blue arrow), and eosinophils (black arrow) **(B)**. Cytokines and chemokines were analyzed in air-pouch lavage from PBS or particles treated mice using Multiplex analysis. Levels of inflammatory proteins IL-6, TNF-α, CCL2, CCL4, CCL11, CXCL1, and CXCL2 are shown **(C)**. Data represent at least five mice per group; error bars denote mean ± SEM. *P < 0.03, **P < 0.009, ***P < 0.0007; Unpaired *t*-test.

Since the *in vitro* studies showed that ZnONWs induced the production of pro-inflammatory cytokines, we examined whether exposure to ZnONWs leads to inflammatory responses *in vivo*. Murine air pouch model was used to detect the local inflammation as described in the methods section. Flow cytometry analysis of the air-pouch lavage showed that instillation of ZnONWs as well as SiONP induced the recruitment of immune cells (Figure 4A and Supplementary Figure 3). The differential counts, as expected from our previous study, revealed that SiONP treated mice induced neutrophilic inflammation but no significant infiltration of macrophages or eosinophils (Figures 4A,B). In contrast, ZnONWs showed significant increase in both macrophages and eosinophils. Although, neutrophil influx was observed by ZnONWs it was relatively weaker compared to SiONP exposed mice (Figures 4A,B). These results suggest that ZnONWs induced inflammation is both qualitatively and quantitatively different from that induced by SiONP. Multiplex analysis of air-pouch lavage from PBS or nanomaterial treated mice was performed to test the levels of various inflammatory proteins detected in the air-pouch. Among the 28 cytokines and chemokines tested by this assay, ZnONWs induced significant increase in IL-6, CCL11, CCL2, TNF-α, and CXCL1 levels, consistent with the cell types recruited into the air-pouch (Figure 4C).

### ZnONWs Induce Eosinophilic Lung Inflammation

**Figure 5:**
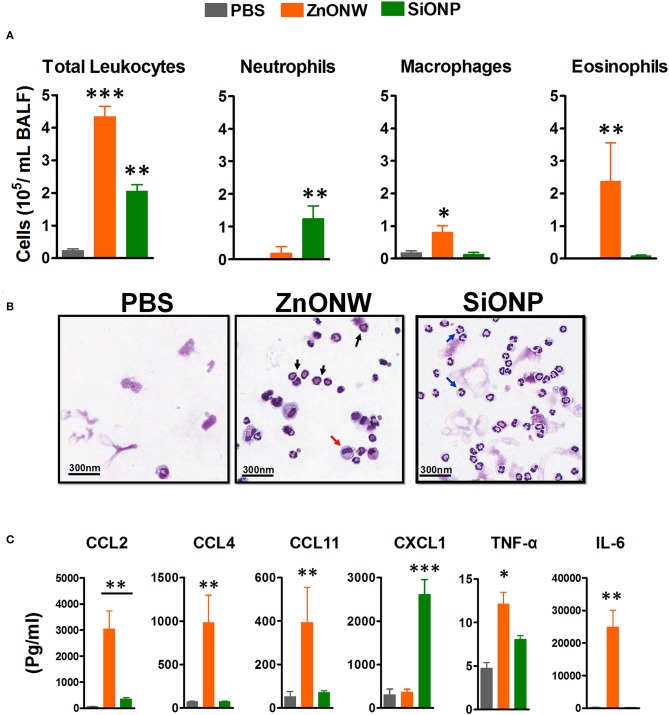
Nano-particle induced acute lung inflammation. WT mice were intratracheal instilled with nanoparticles (ZnONWs or SiONPs) or PBS as described in methods. Lungs were lavaged with 3 ml of buffer to assess infiltrating immune cells as identified by flow cytometry of BALFs **(A)**. Total number of leukocytes and differential count for cell types recruited in the lung are indicated above. Data are representative of at least five mice per group. Representative cytospin slides showing macrophages (red arrow), neutrophils (blue arrow), and eosinophils (black arrow) **(B)**. Cytokines and chemokines levels were analyzed in BALFs from PBS or particles treated mice using Multiplex analysis. Levels of inflammatory proteins IL-6, TNF-α, CCL2, CCL3, CCL4, CCL11, CXCL1, and CXCL2 are shown **(C)**. Data represent at least five mice per group; error bars denote mean ± SEM. *P < 0.03, **P < 0.009, ***P < 0.0007; Unpaired *t*-test.

Previous studies showed evidence for ZnONPs induced lung inflammation in mice and rats (30–32). Therefore, we investigated lung inflammation induced by ZnONWs in an acute 2-day lung inflammation model. Both SiONPs and ZnONWs led to massive influx of leukocytes into the lung (Figure 5A). Flow cytometry analysis of BALFs showed that macrophages and eosinophils were the major cells infiltrated the lung with ZnONWs (Figure 5B and Supplementary Figure 4). In contrast to the air pouch model, where neutrophils were also significantly increased, in lungs only macrophages and eosinophils were elevated upon ZnONWs exposure (Figure 5B). The multiplex analysis of various inflammatory proteins in the BALF showed elevated levels of IL-6, CCL2, CCL4, and CCL11(Figure 5C). These results suggest that ZnONWs activate inflammatory pathways distinct from those induced by SiONPs.

### ZnONWs Upregulate TNF-α, IL-6, CCL11, and CCL2 mRNA Expression

**Figure 6:**
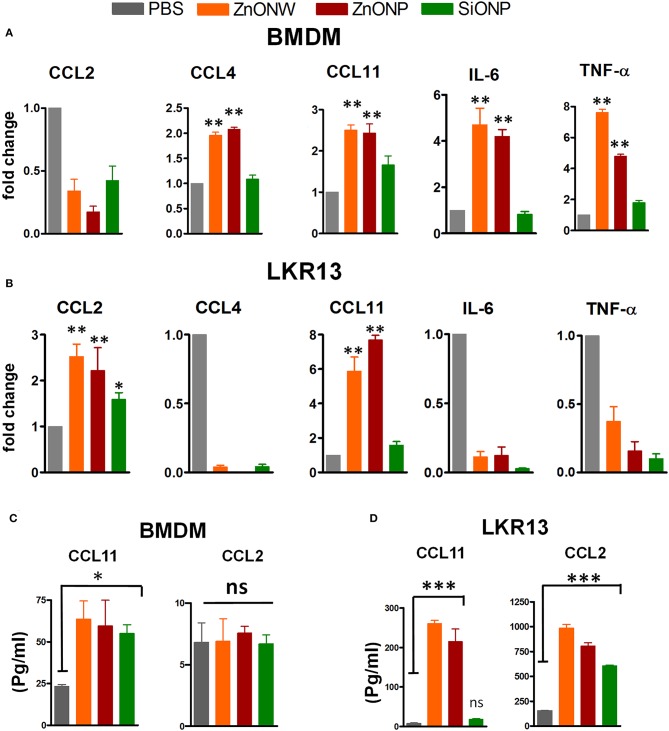
Regulation of inflammatory mediators by ZnONWs exposure. BMDMs were treated with LPS (10 ng/ml) or PBS for 3 h and washed and then cells were treated with nano particles or PBS for 6 h and) were assessed for production of chemokines and cytokines *in vitro*. mRNA levels were analyzed in PBS or particles treated cells using qRT-PCR. Levels of CCL2, CCL4, CCL11, IL-6 (in LPS primed cells) and TNF-α (without LPS priming) transcripts were measured in total RNA and expressed as fold change over PBS treated cells for BMDM **(A)**. Lung epithelial cell line, LKR-13 were treated with nano particles for 6 h with no prior LPS priming and the mRNA levels of indicated chemokines and cytokines was analyzed **(B)** Data are representative of three independent experiments in triplicate cultures and expressed as ± SEM. *P < 0.05, **P <0.01. The levels of CCL11 and CCL2 proteins were determined in the culture supernatants from the experiments in a and b by ELISA **(C,D)**. Data are from one of three experiments in triplicate cultures and expressed as ± SEM. *P < 0.05, ***P <0.0001.

Our previous studies showed that CS exposure in lungs led to an increase in LTB_4_, IL-1β, and many CXC chemokines (29). Since, ZnONWs induced inflammation is significantly different from that of CS-induced inflammation, we further examined the mediators that contribute to this distinct pattern of inflammatory responses. The mRNA levels of various cytokines and chemokines that regulate macrophage and eosinophilic inflammation was measured based on the cell types detected in *in vivo* studies. In BMDMs (Figure 6A) and RAW 246.7 cells (Supplementary Figure 5), the ZnONWs and ZnONPs resulted in significantly increased CCL4, CCL11 and IL-6 and TNF-α expression. In contrast, exposure of lung epithelial cell line, LKR-13 led to enhanced expression of CCL2 and CCL11 mRNAs by ZnONWs and ZnONPs (Figure 6B). The CCL11 protein was also upregulated in culture supernatants of ZnONWs exposed BMDMs and LKR13 cells (Figures 6C,D). Whereas, CCL2 levels increased only in supernatants from LKR13 cells but not in BMDMs (Figures 6C,D).

## Discussion

The results presented here suggest that exposure to ZnONWs could induce inflammatory responses *in vitro* in cultured cells and *in vivo* in mouse models. The soluble Zn ions from the phagolysosomal compartments activate distinct pathways to produce macrophage and eosinophil specific chemokines to recruit these cells to the sites of ZnONWs exposure.

Macrophages play an important role in innate immune response against ENMs (33). The uptake mechanisms of the ENMs largely depend on physicochemical properties of the particles such as shape, size. Previous studies showed that cellular uptake of ENMs such as ZnONPs, gold nanoparticles, sliver nanoparticles, Iron oxide nanoparticles, and carbon nanotubes is facilitated via clathrin, caveolae, and/or scavenger receptor-mediated endocytic pathways (34–40). Our studies showed that ZnONWs were detected in intracellular compartments only in the presence of Baf-A1 (Figures 1B,C) an inhibitor of vacuolar acidification. We surmise that in the absence of Baf-A1, the ZnONWs taken up by these cells are readily solubilized to generate Zn ions. Furthermore, we consistently observed aggregates of the FITC-ZnONWs in the vacuoles (Figure 1B) suggesting these may be taken up by the actin-dependent endocytic pathways. Consistent with this notion, treatment with Cyt.D completely blocked the uptake of FITC-ZnONWs (Figure 1C). Thus, the ZnONWs appear to utilize cellular uptake mechanisms similar to those observed with many other ENMs (34–40). Most ENMs including ZnONPs induce toxicity in cultured cells (12, 33, 41–43). Our results showed that ZnONWs induced toxicity in different cell types including BMDM, RAW 267.4, and LKR13 cells. Significant toxicity was observed at 20 μg/ml of ZnONWs exposure for 18 h. However, at 100 μg/ml the cell viability is further reduced even at 4 h of ZnONWs exposure (Figure 2). These results are in agreement with other previous studies, which suggested that ZnONWs induce toxicity in cultured cells (4, 44). Thus, all further studies with different cell types were performed at 20 μg/ml or less dose of ZnONWs for 6 h exposure.

Inflammatory pathways triggered by ENMs such as SiONPs, titanium dioxide nanoparticles (TiONPs) and carbon nanotubes (CNTs) were all linked to the activation of the inflammasome—caspase-1 related pathway (NLRP3) and release of the cytokine IL-β (45, 46). The current studies show that stimulation of BMDMs and RAW 267.4 cells with ZnONWs did not induce IL-β or LTB_4_ production, whereas SiONPs as expected induced both IL-β and LTB_4_ (Figure 3A). These results suggest that ZnONWs do not activate the NLRP3 inflammasome pathway. However, ZnONPs were known to induce the production of IL-6, and TNF-α (2, 47, 48). Our results also showed that ZnONWs exposure led to a significant increase in the production of both IL-6 and TNF-α in a dose depended manner in RAW267.4 (Supplementary Figure 2) and BMDMs but not in LKR13 (Figure 3B) cells. However, in the presence of Baf-A1 exposure to ZnONWs did not result in any IL-6 or TNF-α production suggesting that soluble Zn ions may be mediating the induction of these cytokines. Interestingly, treatment of cells with ZnCl_2_ did not induce the production of these cytokines (data not shown). A recent study by Muller et al. (4) showed that both ZnONWs and ZnCl_2_ exhibited similar toxicity at all concentrations tested to human monocyte macrophages (HMMs) and they concluded that ZnONWs toxicity is due to the release of ionic Zn^+2^. However, the inflammatory potential of ZnONWs is unknown at concentrations that did not induce cytotoxicity. Interestingly, treatment of cells with ZnCl_2_ did not induce the production of these cytokines. Furthermore, treatment with Baf-A1 or CytD blocked the production of both IL-6 and TNF-α (Figure 3). These data suggested that the uptake and the solubility of particles in the phagolysosomal compartment is needed for the production of IL-6 and TNF-α. Further studies are needed to unravel the mechanisms involved in the location dependent effect of soluble Zn ions in inducing the inflammatory response. Thus, uptake of the particles as well their dissolution within the phagolysosomal compartment appears essential for the induction of cytokines and the externally provided ZnCl_2_ is not sufficient for activating the production of pro-inflammatory cytokines. Furthermore, that both ZnONPs (2, 47, 48) and ZnONWs (current study) induced a similar cytokine profile suggests that it is the chemical nature rather than shape of the ZnO ENMs as the primary cause of the observed cytokine profile. Together, these results suggest that ZnONWs induce a distinct inflammatory pathway from SiONPs and other ENMs.

Skin and inhalation are the most common routes of exposure to ZnONWs. In this study for the first time a murine air-pouch model was used to simulate local exposure to ZnONWs. Earlier studies from our laboratory showed that crystalline silica exposure (CS) induced neutrophilic inflammation in the air pouch model with a significant increase in the levels of IL-1β and LTB_4_ (19). This type of inflammation observed with the CS is linked to the lipidosome activation and LTB_4_ production as well as NLRP3 inflammasome pathway leading to caspase-1 activation (19, 49). In this study, ZnONWs induced inflammation resulted in infiltration of macrophages and eosinophils; production of CCL11, CCL2, IL-6, and TNF-α but not IL-β or LTB_4_. This is in contrast with significant neutrophilic inflammation induced by SiONPs treatment at the site of exposure (Figures 4A,B) and significant levels of IL-β and LTB_4_ in the air-pouch lavage (data not shown). These results strongly support our *in vitro* studies where IL-6 and TNF-α production was induced upon ZnONWs exposure to BMDM and RAW264.7 cells suggesting that ZnONWs do not activate either lipidosome or inflammasome pathways (19, 29). Since IL-6 and TNF-α are produced after ZnONWs exposure, it appears that inflammation induced by ZnONWs is mediated via NF-Kβ pathway.

Previously, the effect of ZnONPs was examined on allergic airway inflammation models (30, 31, 50, 51). In these studies, the highest pulmonary exposure doses of ZnONPs were 1.4–5.4 mg/kg in mice (51) whereas in rats up to 37 mg/kg was used with no mortality or weight reduction (52). Furthermore, these particles were relatively small at 10–50 nm in size leading to rapid dissolution of Zn ions and more toxicity owing to their large surface area. The ZnONWs have a completely different size and shape than spherical ZnONPs and according to Muller et al. ZnONWs appears to have a different dissolution pattern than spherical ZnONPs (4). Since the shape influence the cellular uptake mechanisms (53), we followed the dosing patterns of other ENMs that have similar size and shape to ZnONWs (54–57) and used a 33.5 mg/kg exposure dose in our lung inflammation model. In our study, a massive influx of eosinophils and macrophages in BALFs was observed 2 days after ZnONWs instillation without any prior sensitization (Figures 5A,B). In addition to elevated levels of IL-6 and TNF-α, a significant increase in CCL11 (eotaxin) and CCL2 chemokines is consistent with the cell types detected in BALFs. Previous studies with other ENMs such as TiO2NPs and NiONPs showed eosinophilic inflammation with OVA sensitization or at day 4 post lung instillation, respectively (58, 59). Exposure of rod-like carbon nanotubes (rCNT) also triggered eosinophilic inflammation in wild type mice after 4 h/day for 4 consecutive days of exposure while tangled CNT (tCNT) did not elicit any inflammatory response (60). Our results suggest that the sterile inflammation induced by ZnONWs is distinct from other ENMs such as SiONPs induced inflammation. The major difference relates to the recruitment of eosinophils at the site of exposure by ZnONWs compared to neutrophilic inflammation with SiONPs.

The molecular basis for the *in vivo* ZnONWs mediated cellular infiltration was further analyzed *in vitro* in different cell types. We examined the CCL11, CCL2, IL-6, and TNF-α expression levels in BMDMs (Figure 6A), LKR13 cells (Figure 6B) and RAW 267.4 cells (Supplementary Figures 2, 5) stimulated with ZnONWs. The data suggests that lung epithelial cells and resident macrophages could be the primary responders to ZnONWs producing CCL2 and CCL11 leading to macrophage and eosinophils recruitment to the site of exposure. Moreover, macrophages are considered as one of the major sources for TNF-α production upon activation. When cells are exposed to TNF-α, NF-kβ is activated leading to the expression of many pro- inflammatory cytokines genes such as IL-6 (61). Furthermore, the upregulation of CCL11 and CCL2 mRNA expression is mediated via intracellular signaling of TNF-α through NF-Kβ pathway (62). Since NF-Kβ is a transcription factor known to regulate both CCL11 and CCL2 promoters and signals downstream of both the IL-6 and TNF receptors (62–64). Further studies are needed to define the NF-Kβ signaling pathways that are activated upon ZnONWs exposure involved in the production of CCL11, CLL2, IL-6, and TNF-α.

In summary, this work examined the effect of ZnONWs induced inflammation in a healthy mouse model. ZnONWs induced the production of pro-inflammatory cytokines IL-6 and TNF-α in cultured cells. In mouse models of inflammation, ZnONWs promoted the recruitment of macrophages and eosinophils in to the lung as well as air-pouch. In addition, macrophage specific (CCL2) and eosinophil specific (CCL11) chemokines and the pro- inflammatory cytokines IL-6 and TNF-α were induced in BALFs and air-pouch lavage, consistent with the cell types recruited at the sites of exposure. While these studies clearly outline the mechanisms behind the eosinophil recruitment upon ZnONWs exposure in murine models, the relevance of these observations to human exposure of ZnONWs remains to be established.

## Data Availability Statement

The datasets generated for this study are available on request to the corresponding author.

## Ethics Statement

The animal study was reviewed and approved by University of Louisville Institutional Animal Care and Use Committee (IACUC).

## Author Contributions

RA, SS, SB, MS, and BH provided key intellectual input culminating in the conception and design of the studies presented in this manuscript. The studies were carried out by RA, SS, SB, BH, and VJ. WT and JB provided expertise for cytokine multiplex analysis and FITC labeling studies, respectively. RA, SB, and BH wrote the paper. All authors read and approved the final manuscript.

### Conflict of Interest

MS has a financial interest in Advanced Energy Materials, LLC. The remaining authors declare that the research was conducted in the absence of any commercial or financial relationships that could be construed as a potential conflict of interest.
