# Supplementary material for: Zinc Oxide Nanowires Exposure Induces a Distinct Inflammatory Response via CCL11-Mediated Eosinophil Recruitment
Source: Front Immunol. 2019 Nov 8;10:2604. doi: 10.3389/fimmu.2019.02604 (PMC6856074; doi:10.3389/fimmu.2019.02604)
Supplement: Supplementary file 1 [file Data_Sheet_1.pdf]

Alghsham et al Supplemental Information

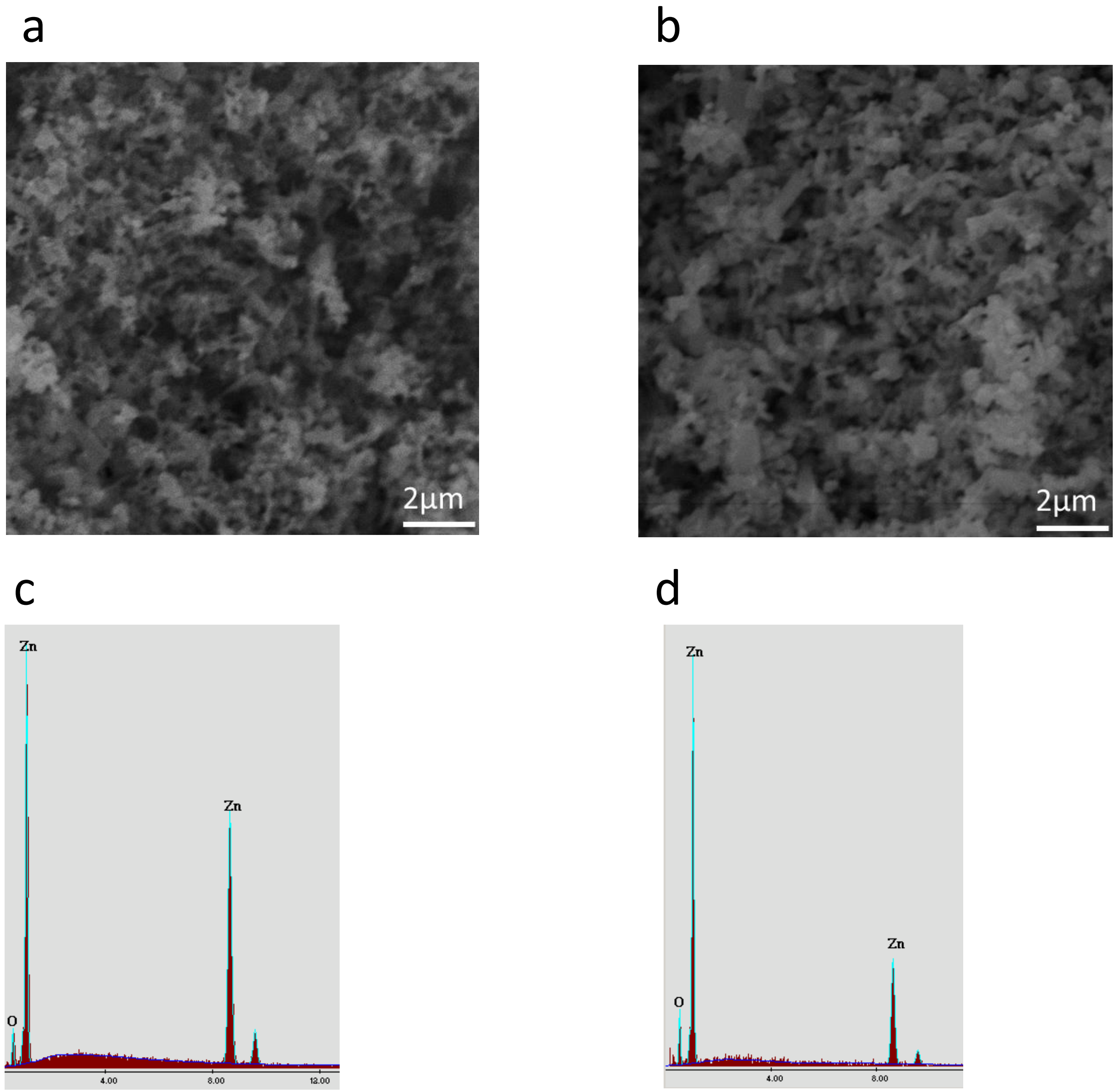

c)

EDAX ZAF Quantification
Element Normalized
SEC Table : Default

Elem	Wt %	At %	K-Ratio
O K	16.10	43.95	0.0457
ZnK	83.90	56.05	0.8105
Total	100.00	100.00	

d)

EDAX ZAF Quantification
Element Normalized
SEC Table : Default

Elem	Wt %	At %	K-Ratio
O K	20.58	51.43	0.0603
ZnK	79.42	48.57	0.7603
Total	100.00	100.00	

Supplementary Figure 1: Heat inactivation of endotoxin did not alter physicochemical properties of ZnONW: ZnONWs were baked overnight as described in methods. Scanning electron micrographs showing the morphology of ZnO nanowire powders – **a**) sample after baking and **b**) as-prepared. The images show similar morphologies for ZnO NW powders in both cases. The elemental analysis is done using energy dispersive spectroscopy within scanning electron microscope (TESCAN VEGA 3 SEM). The results show the presence of only Zn and O in both samples. The quantification (atomic percentage) of the signal indicates slightly Zinc rich nature of baked sample c) compared to as-prepared sample d). In stoichiometric ZnO, the at% ratio should be 50:50.

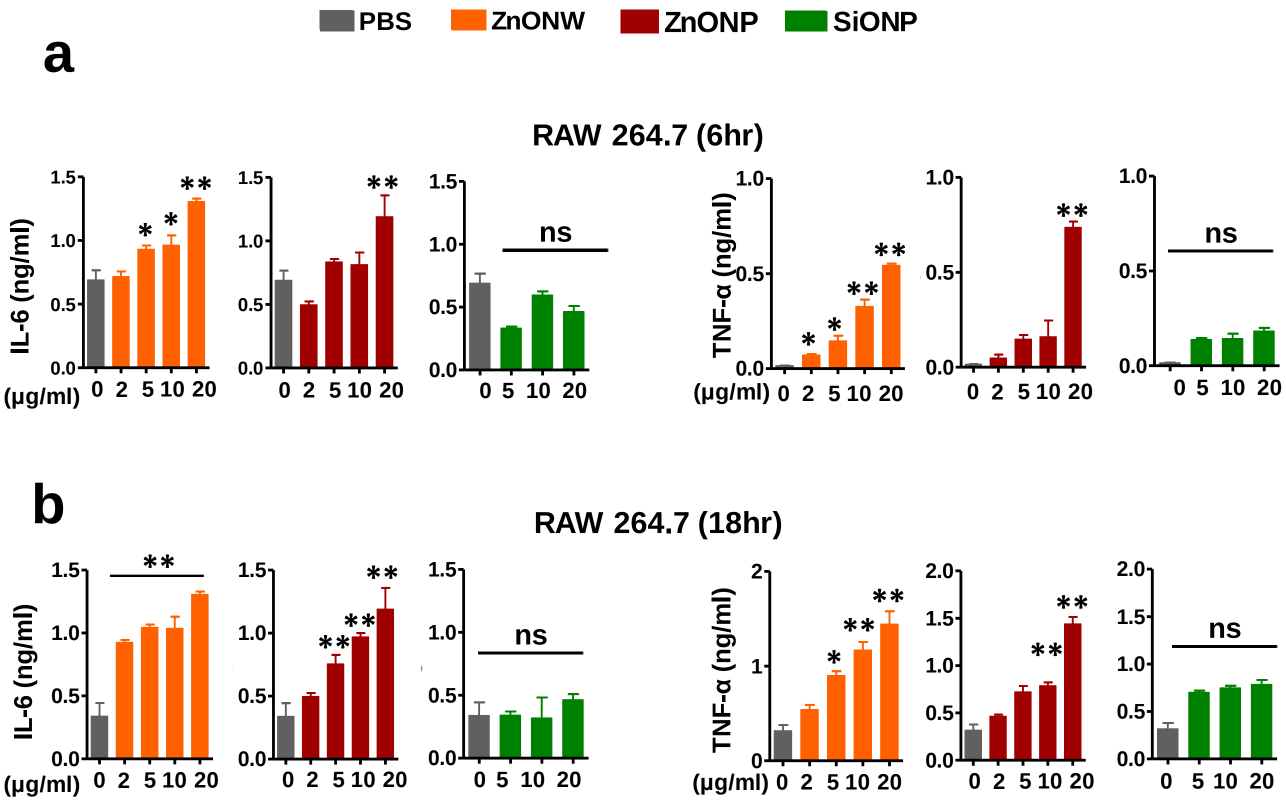

Supplementary Figure 2: ZnONWs exposure induced the release of pro-inflammatory mediators by macrophages *in vitro*.

Pro-inflammatory cytokine levels from PBS or particles (ZnONPs, ZnONWs & SiONPs) treated RAW264.7 cell culture supernatants were analyzed using ELISA. RAW 264.7 cells were primed with LPS (10 ng/ml) or PBS for 3h. Then cells were treated with ZnONWs, ZnONPs, SiONPs or PBS for 6hrs (a) or 18 hrs (b) and the IL-6 (in LPS primed cells) and TNF-α (without LPS priming) levels were measured in the supernatants. Data are representative of three independent experiments in triplicate cultures and values are expressed ± SEM. P=0.05* and =0.005**

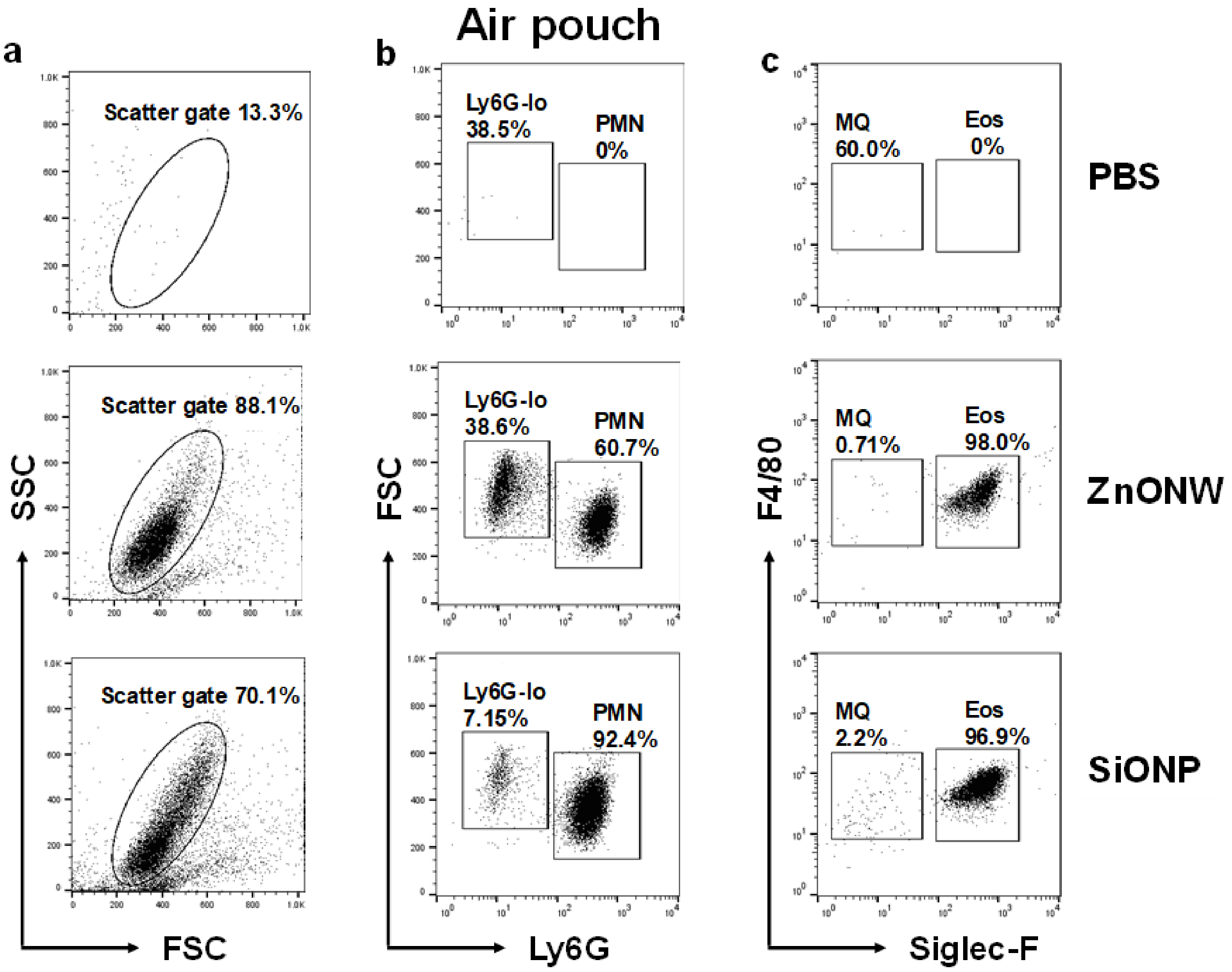

Supplementary figure 3: Identification of various immune cell types in air-pouch lavage fluid by flow cytometry.

In mice treated with ZnONW, SiONP or PBS, lavaged cells were scatter gated (a). On scatter gated cells shown in panel **a**, neutrophils (PMN) were identified as Ly6G^{hi} cells (b). On Ly6G^{lo} gated cells shown in panel **b**, eosinophils (Eos) were identified as Siglec-F^{+} cells and macrophages (MQ) as Siglec-F^{-} F4/80^{+} cells (c).

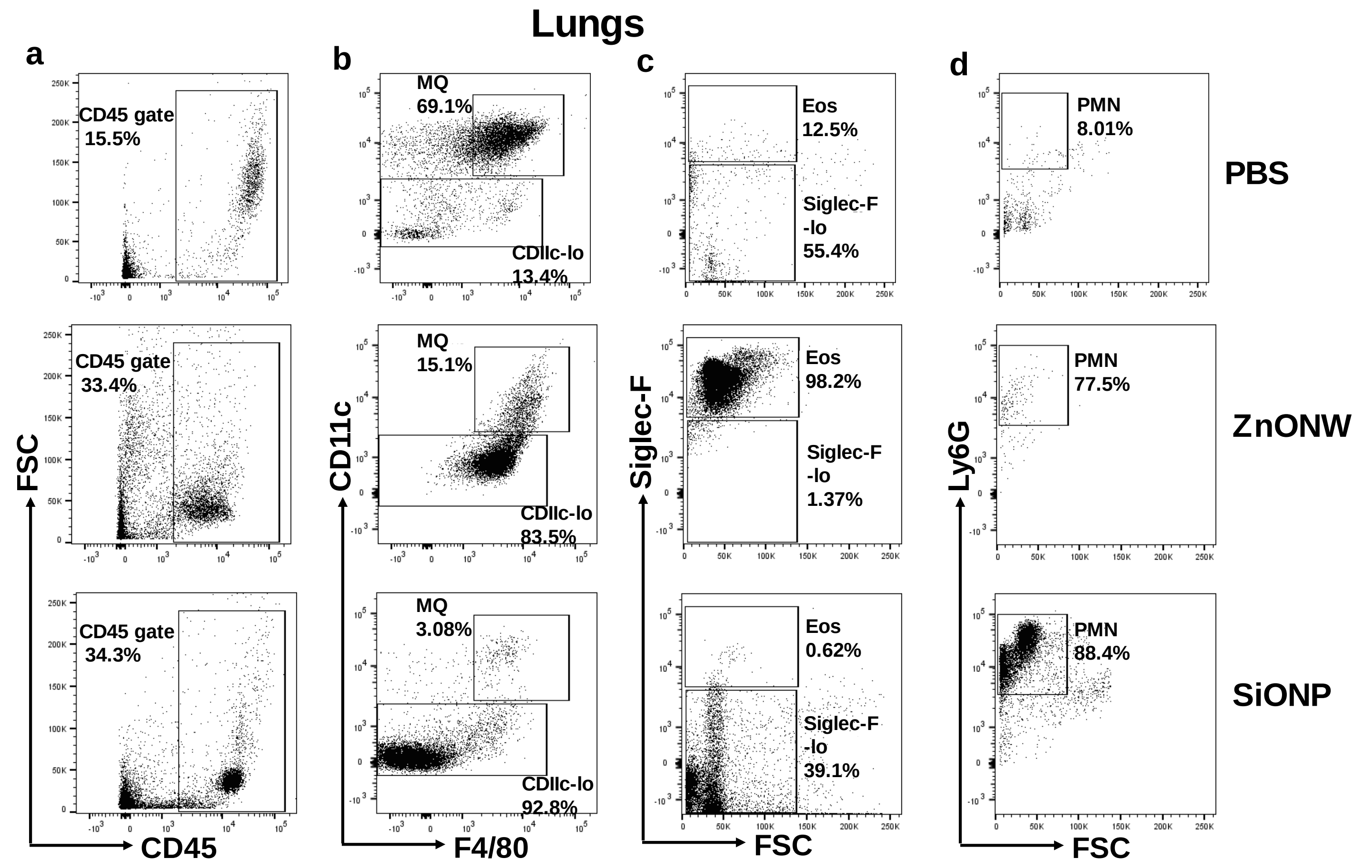

Supplementary figure 4: Identification of various immune cell types in broncho-alveolar lavage fluid by flow cytometry.

In mice treated with ZnONW, SiONP or PBS, lavaged cells were CD45 gated (a). On CD45 gated cells shown in panel **a**, macrophages (MQ) were identified as $CD11c^{hi}$ $F4/80^{+}$ cells (b). On $CD11c^{lo}$ gated cells shown in panel **b**, eosinophils (Eos) were identified as $Siglec\text{-}F^{+}$ cells (c). On $Siglec\text{-}F^{-}$ cells shown in panel **c**, neutrophils (PMN) were identified as $Ly6G^{hi}$ cells (d).

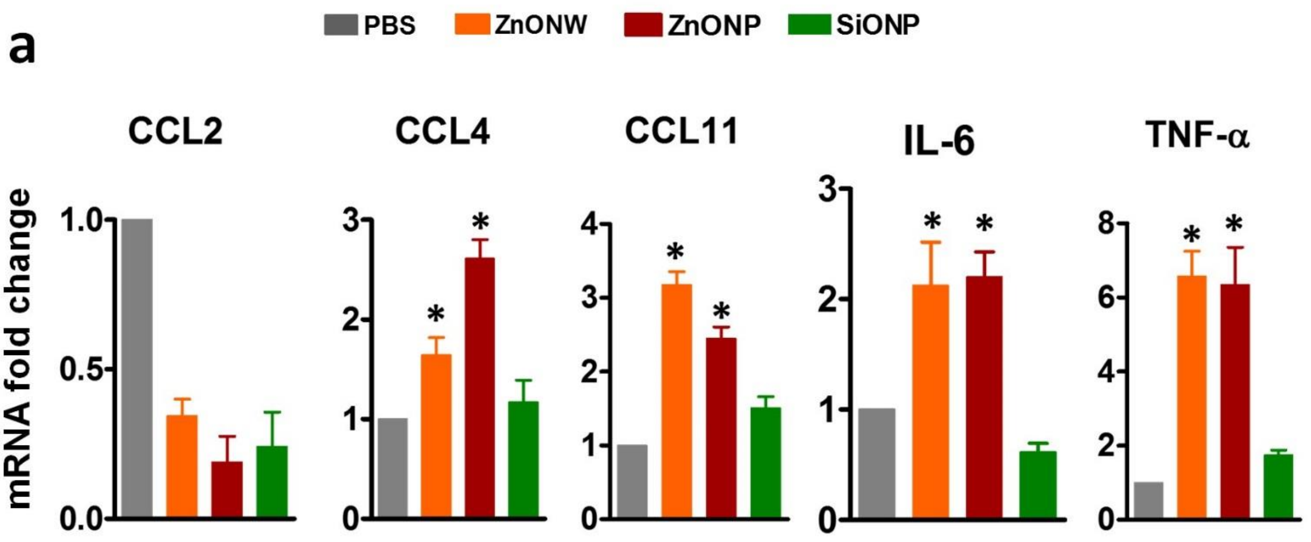

Supplementary Figure 5: Expression levels of inflammatory markers induced by ZnONWs in RAW 264.7 cells.

RAW 264.7 cells exposed to PBS or particles were assessed for production of chemokines and cytokine mRNAs in vitro. CCL2, CCL4, CCL11, IL-6 (in LPS primed cells) and TNF-α (without LPS priming) levels were measured in total RNA and expressed as fold change over PBS treatment. Data are representative of three independent experiments in triplicate cultures and values are expressed fold change ± SEM. *P 0.05
